# Supplementary material for: Carcasses at Fixed Locations Host a Higher Diversity of Necrophilous Beetles
Source: Insects. 2021 May 4;12(5):412. doi: 10.3390/insects12050412 (PMC8147763; doi:10.3390/insects12050412)
Supplement: Supplementary file 1 [file insects-12-00412-s001.zip › insects-1193486-supplementary.pdf]

Supplementary materials

Table S1. Carrion exposition scheme in the Bavarian Forest National Park from June till November 2018.

Plot-ID	Exposition type	Cadaver species	Weight [kg]	Longitude	Latitude	Exposition date [day of the year]	Elevation [masl]
K-01-I	bait place	control (no carcass)	NA	13,41396	48,9375	156	782
K-02-E	random place	control (no carcass)	NA	13,412715	48,9505	156	810
K-03-I	bait place	control (no carcass)	NA	13,217698	49,0987	156	693
K-04-E	random place	control (no carcass)	NA	13,194954	49,1096	156	762
R-01-I	bait place	roe deer	19,1	13,419776	48,9467	156	806
R-02-E	random place	roe deer	25,35	13,393355	48,9491	156	878
R-03-I	bait place	roe deer	14,45	13,202491	49,0995	156	775
R-04-E	random place	roe deer	17,9	13,21265	49,1146	156	808
K-05-I	bait place	control (no carcass)	NA	13,534828	48,8827	184	839
K-06-E	random place	control (no carcass)	NA	13,478374	48,932	184	1052
K-07-I	bait place	control (no carcass)	NA	13,34907	49,0357	184	882
K-08-E	random place	control (no carcass)	NA	13,388033	49,043	184	1125
R-05-I	bait place	roe deer	17,35	13,524246	48,8822	184	851
R-06-E	random place	roe deer	22,75	13,48961	48,9271	184	1081
R-07-I	bait place	roe deer	13,45	13,340252	49,0272	184	825
R-08-E	random place	red deer	87,5	13,375025	49,0396	184	1058
F-13-E	random place	fox	4,85	13,414011	48,9379	219	783
F-14-E	random place	fox	6,4	13,194835	49,112	219	774
K-09-I	bait place	control (no carcass)	NA	13,41278	48,9504	219	809
K-10-E	random place	control (no carcass)	NA	13,403206	48,9649	219	984
K-11-I	bait place	control (no carcass)	NA	13,217698	49,0987	219	693
K-12-E	random place	control (no carcass)	NA	13,218622	49,1074	219	803
R-09-I	bait place	roe deer	14,4	13,41972	48,9467	219	805
R-10-E	random place	roe deer	24,4	13,399378	48,9564	219	933
R-11-I	bait place	roe deer	20	13,202491	49,0995	219	775
R-12-E	random place	roe deer	21	13,224653	49,1136	219	880
K-15-I	bait place	control (no carcass)	NA	13,534828	48,8827	247	839
K-16-E	random place	control (no carcass)	NA	13,478427	48,9319	247	1052
K-17-I	bait place	control (no carcass)	NA	13,349181	49,0358	247	882
K-18-E	random place	control (no carcass)	NA	13,387979	49,043	247	1126
R-15-I	bait place	roe deer	21	13,524246	48,8822	247	851
R-16-E	random place	roe deer	13,5	13,474573	48,9225	247	1003
R-17-I	bait place	roe deer	13,5	13,340252	49,0272	247	825
R-18-E	random place	roe deer	24	13,359511	49,0284	247	1071
F-23-E	random place	fox	4,7	13,396476	48,9351	275	843
F-24-E	random place	fox	6	13,226218	49,091	275	656
K-19-I	bait place	control (no carcass)	NA	13,41278	48,9504	275	809
K-20-E	random place	control (no carcass)	NA	13,326763	48,9323	275	796

K-21-I	bait place	control (no carcass)	NA	13,218663	49,1073	275	802
K-22-E	random place	control (no carcass)	NA	13,231066	49,0844	275	644
R-19-I	bait place	red deer	70	13,419721	48,9467	275	806
R-20-E	random place	red deer	110	13,336821	48,9288	275	791
R-21-I	bait place	roe deer	24	13,202601	49,0996	275	776
R-22-E	random place	roe deer	25	13,222612	49,0763	275	643
F-29-E	random place	fox	7	13,452245	48,9341	310	820
K-25-I	bait place	control (no carcass)	NA	13,534828	48,8827	310	839
K-26-E	random place	control (no carcass)	NA	13,478391	48,932	310	1052
K-27-I	bait place	control (no carcass)	NA	13,34907	49,0357	310	882
K-28-E	random place	control (no carcass)	NA	13,320759	49,0383	310	842
R-25-I	bait place	red deer	30	13,524246	48,8822	310	851
R-26-E	random place	roe deer	18	13,46863	48,9367	310	975
R-27-I	bait place	red deer	90	13,340252	49,0272	310	825
R-28-E	random place	red deer	55	13,336547	49,0349	310	825

In Plot-ID: R stands for roe- or red deer, F stands for fox carcasses, K means control, I stands for intensive (bait place), E stands for extensive (random place); Weight = unfrozen weight at the time of exposition (day 0); masl = meters above sea level.

Table S2. Asymptotic beetle diversity estimates along with related statistics for Hill numbers q = 0, q = 1 and q = 2.

Site (exposition type + decay stage)	Diversity	Observed diversity	Estimator	S.E.	LCL	UCL
Early succession extensive	Species richness	29,00	37,16	8,27	30,57	71,37
Early succession extensive	Shannon diversity	8,25	8,47	0,47	8,25	9,40
Early succession extensive	Simpson diversity	4,66	4,68	0,24	4,66	5,14
Middle succession extensive	Species richness	40,00	55,12	12,47	43,69	102,03
Middle succession extensive	Shannon diversity	6,42	6,48	0,19	6,42	6,85
Middle succession extensive	Simpson diversity	3,21	3,21	0,09	3,21	3,39
Late succession extensive	Species richness	48,00	64,90	12,72	52,54	110,83
Late succession extensive	Shannon diversity	7,99	8,07	0,20	7,99	8,45
Late succession extensive	Simpson diversity	3,86	3,86	0,10	3,86	4,06
Early succession intensive	Species richness	39,00	62,97	20,18	44,71	139,60
Early succession intensive	Shannon diversity	11,58	12,02	0,61	11,58	13,22
Early succession intensive	Simpson diversity	6,53	6,57	0,34	6,53	7,23
Middle succession intensive	Species richness	47,00	65,74	13,07	52,46	111,39
Middle succession intensive	Shannon diversity	12,67	12,90	0,32	12,67	13,53
Middle succession intensive	Simpson diversity	8,92	8,96	0,22	8,92	9,39
Late succession intensive	Species richness	45,00	64,59	14,35	50,42	115,80
Late succession intensive	Shannon diversity	10,85	10,99	0,26	10,85	11,49
Late succession intensive	Simpson diversity	6,87	6,89	0,18	6,87	7,23

In Site: ‘extensive’ stands for random site, ‘intensive’ stands for bait place, ‘early succession’ = fresh stage, ‘middle succession’ = bloated and post-bloating stages and ‘late succession’ = advanced decay and dry remains; Estimator stands for asymptotic estimates; S.E. = estimated bootstrap standard error; LCL & UCL stand for lower and upper confidence limits (95% confidence intervals) for Hill numbers of order q = 0 (Species richness), q = 1 (Shannon diversity) and q = 2 (Simpson diversity).

Table S3. List of 77 species of necrophilous beetles captured at wildlife carcasses, with their indicator value (IndVal), the adjusted *p*-value [1] and the group of sites for which they are characteristic. Significant indicator species are marked in bold.

**Species**	**Family**	**Group of sites**	**IndVal**	**Adjusted *p***
Acrotona parvula	Staphylinidae	random site	0,106	1,000
Anaspis rufilabris	Scraptiidae	random site	0,106	1,000
Anoplotrupes stercorosus	Geotrupidae	random site	0,723	0,181
Anotylus rugosus	Staphylinidae	bait place	0,122	0,785
Anotylus sculpturatus	Staphylinidae	bait place	0,192	0,785
Anthobium melanocephalum	Staphylinidae	bait place	0,122	0,785
Ammoecius brevis	Scarabaeidae	bait place	0,208	0,785
Nimbus contaminatus	Scarabaeidae	random site	0,184	0,785
Acrossus depressus	Scarabaeidae	random site	0,325	0,785
Planolinus fasciatus	Scarabaeidae	random site	0,106	1,000
Teuchestes fossor	Scarabaeidae	bait place	0,122	0,785
Calamosternus granarius	Scarabaeidae	bait place	0,122	0,785
Acrossus luridus	Scarabaeidae	random site	0,106	1,000
Limarus maculatus	Scarabaeidae	random site	0,193	0,959
Melinopterus prodromus	Scarabaeidae	bait place	0,230	0,785
Acrossus rufipes	Scarabaeidae	bait place	0,358	0,785
Melinopterus sphacelatus	Scarabaeidae	bait place	0,122	0,785
Volinus sticticus	Scarabaeidae	bait place	0,122	0,785
Arpedium quadrum	Staphylinidae	random site	0,158	1,000
Atheta excellens	Staphylinidae	random site	0,106	1,000
Atheta longicornis	Staphylinidae	bait place	0,122	0,785
Atrecus affinis	Staphylinidae	bait place	0,122	0,785
Creophilus maxillosus	Staphylinidae	bait place	0,398	0,785
Geotrupes stercorarius	Geotrupidae	bait place	0,122	0,785
Hister unicolor	Histeridae	random site	0,106	1,000
Lampyris noctiluca	Lampyridae	random site	0,106	1,000
Lordithon trinotatus	Staphylinidae	random site	0,306	0,625
Margarinotus brunneus	Histeridae	bait place	0,122	0,785
Margarinotus striola	Histeridae	bait place	0,484	0,625
Megarthrus depressus	Staphylinidae	bait place	0,122	0,785
Necrophilus subterraneus	Agyrtidae	bait place	0,229	0,713
Omalium rivulare	Staphylinidae	random site	0,240	1,000
Omalium rugatum	Staphylinidae	bait place	0,122	0,785
Omalium septentrionis	Staphylinidae	bait place	0,358	0,959
Ontholestes tessellatus	Staphylinidae	random site	0,310	1,000
Oxypoda formosa	Staphylinidae	random site	0,106	1,000
Oxypoda opaca	Staphylinidae	bait place	0,122	0,785
Oxytelus laqueatus	Staphylinidae	random site	0,258	0,785
Philonthus addendus	Staphylinidae	random site	0,106	1,000
Philonthus carbonarius	Staphylinidae	random site	0,506	1,000
Philonthus fimetarius	Staphylinidae	random site	0,497	1,000

Philonthus laevicollis	Staphylinidae	random site	0,194	1,000
Philonthus marginatus	Staphylinidae	random site	0,241	0,959
Philonthus pseudovarians	Staphylinidae	bait place	0,122	0,785
Philonthus rufipes	Staphylinidae	bait place	0,215	1,000
Philonthus splendens	Staphylinidae	random site	0,106	1,000
Proteinus brachypterus	Staphylinidae	bait place	0,122	0,785
Quedius cinctus	Staphylinidae	random site	0,319	0,785
Rugilus mixtus	Staphylinidae	bait place	0,122	0,785
Rugilus rufipes	Staphylinidae	bait place	0,302	0,785
Saprinus semistriatus	Histeridae	bait place	0,408	0,785
Sphaerites glabratus	Sphaeritidae	random site	0,201	1,000
Tachinus pallipes	Staphylinidae	random site	0,619	0,181
Tachyporus pusillus	Staphylinidae	random site	0,106	1,000
Xantholinus longiventris	Staphylinidae	bait place	0,123	1,000
Xantholinus tricolor	Staphylinidae	bait place	0,122	0,785
Aleochara curtula	Staphylinidae	bait place	0,147	0,959
Apocatops nigrita	Leiodidae	random site	0,106	1,000
Catops kirbyi	Leiodidae	bait place	0,092	1,000
Dermestes laniarius	Dermestidae	bait place	0,122	0,785
Dermestes murinus	Dermestidae	random site	0,116	1,000
Necrobia rufipes	Cleridae	bait place	0,173	0,980
Necrobia violacea	**Cleridae**	**bait place**	**0,540**	**0,004**
Necrodes littoralis	Silphidae	bait place	0,411	0,959
Nicrophorus humator	Silphidae	bait place	0,431	0,785
Nicrophorus interruptus	Silphidae	random site	0,189	0,785
Nicrophorus investigator	Silphidae	random site	0,385	0,980
Nicrophorus vespillo	Silphidae	random site	0,150	1,000
Nicrophorus vespilloides	Silphidae	random site	0,530	0,959
Oiceoptoma thoracicum	Silphidae	bait place	0,447	0,959
Omosita depressa	**Nitidulidae**	**bait place**	**0,662**	**0,004**
Ontholestes murinus	Staphylinidae	bait place	0,212	0,625
Sciodrepoides fumatus	Leiodidae	random site	0,184	0,785
Sciodrepoides watsoni	Leiodidae	random site	0,106	1,000
Thanatophilus rugosus	Silphidae	bait place	0,406	0,181
Thanatophilus sinuatus	Silphidae	bait place	0,542	0,181
Trox scaber	Trogidae	bait place	0,122	0,785

Table S4. List of 57 species of necrophilous beetles captured at wildlife carcasses, with their indicator value (IndVal), the adjusted *p*-value [1] and the group of sites for which they are characteristic (see Figure 4 in the main manuscript for code explanations). Significant indicator species are marked in bold.

Species	Family	Group of sites	IndVal	Adjusted *p*
Acrotona parvula	Staphylinidae	3+6+9	0,149	1,000
Anaspis rufilabris	Scraptiidae	2+5+8	0,200	0,977
Anoplotrupes stercorosus	**Geotrupidae**	**2+3+5+6+8+9**	**0,866**	**0,017**
Anotylus sculpturatus	Staphylinidae	1+2+3	0,218	0,872
Ammoecius brevis	Scarabaeidae	1+4+7	0,324	0,219
Nimbus contaminatus	Scarabaeidae	4+5+6	0,236	0,977
Acrossus depressus	Scarabaeidae	7+8+9	0,458	0,245
Planolinus fasciatus	Scarabaeidae	1+4+7	0,229	0,621
Acrossus luridus	Scarabaeidae	3+6+9	0,149	1,000
Limarus maculatus	Scarabaeidae	1+2+4+5+7+8	0,337	0,381
Melinopterus prodromus	Scarabaeidae	7+8+9	0,267	0,381
Acrossus rufipes	Scarabaeidae	7+8+9	0,496	0,211
Arpedium quadrum	Staphylinidae	7+8+9	0,324	0,381
Atheta excellens	Staphylinidae	3+6+9	0,149	1,000
Creophilus maxillosus	Staphylinidae	2+3+5+6+8+9	0,521	0,211
Hister unicolor	Histeridae	2+5+8	0,200	0,977
Lampyris noctiluca	Lampyridae	3+6+9	0,149	1,000
Lordithon trinotatus	Staphylinidae	1+2+4+5+7+8	0,381	0,457
Margarinotus striola	Histeridae	2+3+5+6+8+9	0,515	0,346
Necrophilus subterraneus	Agyrtidae	3+6+9	0,149	1,000
Omalium rivulare	Staphylinidae	1+4+7	0,433	0,219
Omalium septentrionis	Staphylinidae	1+2+4+5+7+8	0,546	0,117
Ontholestes tessellatus	Staphylinidae	2+3+5+6+8+9	0,493	0,219
Oxypoda formosa	Staphylinidae	1+2+3	0,218	0,872
Oxytelus laqueatus	**Staphylinidae**	**7+8+9**	**0,568**	**0,017**
Philonthus addendus	Staphylinidae	3+6+9	0,149	1,000
Philonthus carbonarius	Staphylinidae	2+3+5+6+8+9	0,661	0,219
Philonthus fimetarius	Staphylinidae	2+3+5+6+8+9	0,701	0,098
Philonthus laevicollis	Staphylinidae	2+3+5+6+8+9	0,234	1,000
Philonthus marginatus	**Staphylinidae**	**7+8+9**	**0,629**	**0,011**
Philonthus rufipes	Staphylinidae	2+3+5+6+8+9	0,272	1,000
Philonthus splendens	Staphylinidae	2+5+8	0,200	0,977
Quedius cinctus	Staphylinidae	1+2+4+5+7+8	0,421	0,621
Rugilus rufipes	Staphylinidae	2+3+5+6+8+9	0,208	1,000
Saprinus semistriatus	Histeridae	2+3+5+6+8+9	0,521	0,211
Sphaerites glabratus	Sphaeritidae	4+5+6	0,266	1,000
Tachinus pallipes	Staphylinidae	2+3+5+6+8+9	0,676	0,457
Tachyporus pusillus	Staphylinidae	3+6+9	0,149	1,000
Xantholinus longiventris	Staphylinidae	4+5+6	0,192	1,000
Aleochara curtula	Staphylinidae	2+5+8	0,200	0,977

Apocatops nigrita	Leiodidae	3+6+9	0,149	1,000
Catops kirbyi	Leiodidae	3+6+9	0,149	1,000
Dermestes murinus	Dermestidae	3+6+9	0,211	0,999
Necrobia rufipes	Cleridae	7+8+9	0,255	0,457
Necrobia violacea	Cleridae	3+6+9	0,149	1,000
Necrodes littoralis	Silphidae	2+3+5+6+8+9	0,598	0,098
Nicrophorus humator	Silphidae	2+3+5+6+8+9	0,535	0,353
Nicrophorus interruptus	Silphidae	2+3+5+6+8+9	0,239	1,000
Nicrophorus investigator	Silphidae	2+3+5+6+8+9	0,499	0,649
Nicrophorus vespillo	Silphidae	3+6+9	0,200	1,000
Nicrophorus vespilloides	Silphidae	1+2+4+5+7+8	0,747	0,098
Oiceoptoma thoracicum	Silphidae	2+3+5+6+8+9	0,613	0,211
Omosita depressa	Nitidulidae	4+5+6	0,385	0,372
Sciodrepoides fumatus	Leiodidae	4+5+6	0,236	0,977
Sciodrepoides watsoni	Leiodidae	1+2+3	0,218	0,872
Thanatophilus rugosus	Silphidae	7+8+9	0,421	0,211
Thanatophilus sinuatus	Silphidae	2+3+5+6+8+9	0,598	0,117

Table S5. List of 62 species of necrophilous beetles captured at wildlife carcasses, with their indicator value (IndVal), the adjusted *p*-value [1] and the group of sites for which they are characteristic (see Figure 4 in the main manuscript for code explanations; no small (fox) cadavers were exposed at bait places). Significant indicator species are marked in bold.

Species	Family	Group of sites	IndVal	Adjusted *p*
Anoplotrupes stercorosus	Geotrupidae	5+6+8+9	0,847	0,306
Anotylus rugosus	Staphylinidae	6+9	0,169	1,000
Anotylus sculpturatus	Staphylinidae	5+8	0,447	0,170
Anthobium melanocephalum	Staphylinidae	7+8+9	0,316	0,422
Ammoecius brevis	Scarabaeidae	4+5+7+8	0,296	0,693
Acrossus depressus	Scarabaeidae	4+5+6	0,350	0,923
Teuchestes fossor	Scarabaeidae	6+9	0,169	1,000
Calamosternus granarius	Scarabaeidae	6+9	0,169	1,000
Limarus maculatus	Scarabaeidae	7+8+9	0,239	0,956
Melinopterus prodromus	**Scarabaeidae**	**7+8+9**	**0,545**	**0,012**
Acrossus rufipes	Scarabaeidae	4+5+6	0,513	0,590
Melinopterus sphacelatus	Scarabaeidae	7+8+9	0,316	0,422
Volinus sticticus	Scarabaeidae	7+8+9	0,316	0,422
Arpedium quadrum	Staphylinidae	7+8+9	0,415	0,306
Atheta longicornis	Staphylinidae	6+9	0,169	1,000
Atrecus affinis	Staphylinidae	5+8	0,258	0,612
Creophilus maxillosus	Staphylinidae	5+6+8+9	0,600	0,306
Geotrupes stercorarius	Geotrupidae	6+9	0,169	1,000
Lordithon trinotatus	Staphylinidae	7+8+9	0,447	0,218
Margarinotus brunneus	Histeridae	5+8	0,258	0,612
Margarinotus striola	Histeridae	5+6+8+9	0,622	0,510
Megarthrus depressus	Staphylinidae	4+7	0,243	0,693
Necrophilus subterraneus	Agyrtidae	4+5+7+8	0,287	0,719
Omalium rivulare	Staphylinidae	7+8+9	0,518	0,218
Omalium rugatum	Staphylinidae	4+7	0,243	0,693
Omalium septentrionis	Staphylinidae	7+8+9	0,576	0,306
Ontholestes tessellatus	Staphylinidae	5+8	0,517	0,306
Oxypoda opaca	Staphylinidae	4+7	0,243	0,693
Oxytelus laqueatus	Staphylinidae	4+5+7+8	0,235	0,967
Philonthus carbonarius	Staphylinidae	4+5+6	0,761	0,170
Philonthus fimetarius	Staphylinidae	4+5+7+8	0,642	0,663
Philonthus laevicollis	Staphylinidae	5+6+8+9	0,283	0,888
Philonthus marginatus	Staphylinidae	4+5+6	0,296	0,956
Philonthus pseudovarians	Staphylinidae	5+8	0,258	0,612
Philonthus rufipes	Staphylinidae	5+6+8+9	0,346	0,693
Proteinus brachypterus	Staphylinidae	7+8+9	0,316	0,422
Quedius cinctus	Staphylinidae	4+7	0,423	0,444
Rugilus mixtus	Staphylinidae	4+7	0,243	0,693
Rugilus rufipes	Staphylinidae	4+5+7+8	0,458	0,422
Saprinus semistriatus	Histeridae	4+5+6	0,562	0,444

Sphaerites glabratus	Sphaeritidae	4+5+7+8	0,231	0,996
Tachinus pallipes	Staphylinidae	5+6+8+9	0,567	0,956
Xantholinus longiventris	Staphylinidae	5+8	0,220	0,967
Xantholinus tricolor	Staphylinidae	5+8	0,258	0,612
Aleochara curtula	Staphylinidae	4+5+7+8	0,250	0,663
Catops kirbyi	Leiodidae	6+9	0,169	1,000
Dermestes laniarius	Dermestidae	6+9	0,169	1,000
Dermestes murinus	Dermestidae	5+8	0,258	0,612
Necrobia rufipes	Cleridae	5+6+8+9	0,245	0,923
Necrobia violacea	Cleridae	5+6+8+9	0,593	0,403
Necrodes littoralis	Silphidae	5+6+8+9	0,613	0,344
Nicrophorus humator	Silphidae	4+5+7+8	0,646	0,306
Nicrophorus interruptus	Silphidae	5+8	0,258	0,612
Nicrophorus investigator	Silphidae	4+5+7+8	0,622	0,218
Nicrophorus vespillo	Silphidae	5+8	0,335	0,422
Nicrophorus vespilloides	Silphidae	4+5+7+8	0,782	0,087
Oiceoptoma thoracicum	Silphidae	4+5+6	0,635	0,344
Omosita depressa	Nitidulidae	4+5+7+8	0,695	0,403
Ontholestes murinus	Staphylinidae	5+6+8+9	0,245	0,923
Thanatophilus rugosus	Silphidae	4+5+6	0,496	0,612
Thanatophilus sinuatus	Silphidae	5+6+8+9	0,644	0,612
Trox scaber	Trogidae	4+7	0,243	0,693

References

1. Cáceres, M. De; Legendre, P. Associations between species and groups of sites: indices and statistical inference. *Ecology* **2009**, *90*, 3566–3574.
